# Low back pain and FokI (rs2228570) polymorphism of vitamin D receptor in athletes

**DOI:** 10.1186/s13102-017-0069-x

**Published:** 2017-02-07

**Authors:** Sabina Cauci, Francesca Migliozzi, Carlo Simone Trombetta, Ilaria Venuto, Paola Saccheri, Luciana Travan, Giovanni Chiriacò

**Affiliations:** 1 Department of Medical and Biological Sciences, School of Medicine, University of Udine, Udine, 33100 Italy; 2 Department of Medicine, Surgery and Health Sciences, School of Medicine, University of Trieste, Trieste, 34100 Italy; 3 Department of Experimental and Clinical Medical Sciences, School of Medicine, University of Udine, Udine, 33100 Italy; 4 Clinical Biochemistry and Molecular Biology, Department of Medical and Biological Sciences, Piazzale Kolbe 4, 33100 Udine, Italy

**Keywords:** Lumbar pain, Exercise, Vitamin D receptor, Vitamin D receptor gene, Vitamin D receptor polymorphism, FokI polymorphism, Exposure to vibrations, Smoking and low back pain, Discopathies

## Abstract

**Background:**

Low back pain (LBP) is common in athletes. LBP can be detrimental to athletic performance and health. Factors predisposing to LBP in athletes remain elusive and require further studies. We investigated whether carriage of a specific genotype and/or allele of vitamin D receptor gene (VDR) FokI polymorphism (rs2228570) was a risk factor for LBP in athletes of different sports disciplines.

**Methods:**

This genotype/phenotype association case-control study included 60 Italian athletes (25 females and 35 males; mean age 33.9 ± 13.3 years; body-mass-index 23.5 ± 3.5 kg/m^2^) of which 16.7% were swimmers, 11.7% soccer players, 11.7% volleyball players, 10.0% rugby players and other disciplines. *VDR*-FokI polymorphism was measured by PCR-RFLP in 24 athletes with LBP and 36 athletes without LBP episodes. Absence or presence of the FokI restriction site was denoted “F” and “f”, respectively. Other risk factors were evaluated by a questionnaire.

**Results:**

The homozygous FF genotype was found in 58.3% (14/24) of athletes with LBP versus 27.8% (10/36) of athletes without LBP, adjusted OR = 5.78, 95% CI 1.41–23.8, *P* = 0.015. The F allele was a 2-fold risk factor to develop LBP, adjusted OR = 2.55, 95% CI 1.02–6.43, P = 0.046, while f allele was protective. Exposure to vehicle vibrations ≥2 h daily, and family history of lumbar spine pathology were significant risk factors for LBP with OR = 3.54, and OR = 9.21, respectively.

**Conclusions:**

This is the first study in which an association between *VDR*-FokI polymorphism and LBP in athletes was found. Further research is needed to extend our results, and to clarify the biochemical pathways associated with how vitamin D modulates LBP in athletes. The *VDR-*FokI polymorphism should be considered when developing genetic focused studies of precision medicine on health in athletes.

**Electronic supplementary material:**

The online version of this article (doi:10.1186/s13102-017-0069-x) contains supplementary material, which is available to authorized users.

## Background

Low back pain (LBP) can have a significant impact on athletes’ performance and may sometimes cause disability [1–3]. LBP has been largely studied in the general population; approximately 50–80% of people have at least one episode during their lifetime [4–7]. Conversely, there are few studies focused on LBP in athletes. In studies were lumbar spine pain is evaluated as a symptom by questionnaire, it had a prevalence of around 30–40% [1, 2, 8]. Prevalence of LBP seems to vary according to type of sport discipline [9], rates over 70% were reported for bowling/skittles, canoe, cycling, gymnastics, rowing, shooting, snowboarding, and volleyball [8]. A recent Iranian study [10] among college female athletes found a life-time prevalence of LBP of nearly 70% in basketball, karate, and track-and-field athletes.

LBP has a complex etiology and it is often recurrent [11–13]. In the general population many environmental/behavioral risk factors, including age, sex, weight, occupational load, smoking and exposure to vehicle vibration were demonstrated to contribute to spinal degeneration and severe pain [7, 14, 15]. In athletes, exposure to high exercise load [16], decreased lumbar flexion and extension, shoulder flexibility, and forward bending have been involved in LBP and disc degeneration processes [1, 9, 17]. Recently, some epidemiologic and genetic studies [6, 7, 14, 15, 18–20] performed in the general non-athletic population supported the notion that LBP and disc degeneration disorders may be affected by genetic polymorphisms of the vitamin D receptor gene (*VDR*) including FokI, BsmI, TaqI and ApaI single nucleotide polymorphisms (SNPs).

Among several SNPs identified in the *VDR* gene, only the FokI polymorphism is located in an exon sequence [21, 22]. The FokI polymorphism (rs2228570) is a C to T transition polymorphic site located in the *VDR* start codon, affecting the amino acid sequence and the function of the encoded receptor protein [21]. The allelic variants of this polymorphism code for structurally different receptor proteins (from a 424 aminoacids wild type encoded by the F allele to a 427 amino acid long protein encoded by the f allele). The short and long protein variants are associated with a different efficiency of *VDR* binding with the transcription factor II B (TFIIB) and, thus, to a different ability to induce transcription of VDR-dependent genes (vitamin D response elements, VDREs [23]). The shorter wild protein (corresponding to the F allele) appears to interact more efficiently with TFIIB showing a higher transcriptional rate [24, 25]. Consequently, studies concerning the possible association of *VDR*-FokI polymorphism with LBP and disc degeneration may be particularly interesting for the potential biological significance. *VDR*-FokI is an independent polymorphic site, not in linkage disequilibrium with other *VDR*-SNPs [21]. Distribution of *VDR-*FokI polymorphism genotypes and alleles can vary with the genetic backgrounds. Therefore, specific ethnic group focused studies are warranted [6].

Wide evidence supports the notion that the vitamin D endocrine system, including active vitamin D hormone (1α,25-dihydroxyvitamin D_3_), its receptor and the enzymes involved in the generation of the biologically-active forms of vitamin D are implicated in the modulation of different biological processes, including skeletal metabolism, immunological response (in general, vitamin D/VDR action promotes interleukin-1 and innate immune response, while it attenuates adaptive immunity), detoxification, oxidative stress, cancer-related metabolic pathways, proliferation and differentiation of a wide variety of cell types [26, 27]. Recently, the presence of VDR was evident in skeletal muscle [28, 29] and also in intervertebral disc cells, more specifically in the nucleus pulposus and annulus fibrosus cells, which constitute the two different major parts of the intervertebral disc [30]. This highlighted that biological interactions of intervertebral disc cells with the vitamin D metabolites may be crucial to disc health, consequently an altered vitamin D signaling could have a role in the pathophysiology of the disc degeneration and LBP [6].

To our knowledge, there are no studies investigating the association of the *VDR*-FokI polymorphism and LBP in athletes. This polymorphism by changing the sequence and activity of the VDR protein could affect the activity of the wide variety of genes modulated by the VDR nuclear receptor. Identification of genetic traits in athletes predisposing to LBP might help clinicians, coaches, sport trainers, and athletes themselves to develop personalized strategies to prevent or reduce LBP, for example, by modification of some lifestyles habits and/or kind of training.

The aims of this study were to evaluate the *VDR*-FokI genotypic and allelic frequencies distribution in athletes with LBP in comparison with asymptomatic athletes (no-LBP), and to analyze the interplay of genetic and behavioral/environmental factors in the development of LBP in athletes.

## Methods

### Population

Study participants were enrolled consecutively for an observational study approved by the Local Institutional Ethical Committee of each participating institution, in accordance with Declaration of Helsinki. All participants signed a written informed consent before entering the study.

Italian white unrelated athletes (age range 18–60 years) regularly performing 5 or more hours weekly of sport activity, including training and competitions, were recruited as volunteers into our study. A blood sample was requested and a survey was conducted through a questionnaire. Recruitment was performed through announcements at the Sport Sciences Campus in Gemona of Udine University. Before being enrolled into the study, each athlete was interviewed to assess whether he or she fulfilled the following inclusion criteria: a) no surgery for lumbar pathology; b) no acute severe traumatic events such as a car accident that damaged the spine or because of a contact injury in sport or due to poor biomechanics; c) no documented medical history of spondylolistesis (because this condition has specific characteristics different from other common causes of LBP [7, 15, 31, 32]; d) absence of any acute or chronic disease (like scoliosis, cervical diseases, autoimmune diseases, rheumatoid arthritis, osteoporosis, fibromyalgia, diabetes mellitus, cardiovascular disease, or tumors etc.); e) no use of anabolic steroids or other doping substances; f) no use of vitamin D supplementation; and g) no pregnancy at study entry [7, 14, 33]. For inclusion in the LBP cases subjects had to answer “yes” to the following questions: “Have you had in your life low back pain for more than 1 day?”. “If yes, was this low back pain bad enough to limit your usual activities, or change your daily routine, or sports activities including absenteeism from exercise training sessions, competitions, absence from work and/or class attending for more than 1 day (not due to menstruation)?” [7, 10, 34]. For inclusion in the control group (no-LBP) athletes had to answer “no” to the question “Have you had in your life low back pain for more than 1 day?”. In other words, controls have never had episodes of low back pain lasting for more than 1 day lifelong. Each participant completed a questionnaire assessing demographic characteristics, medical history, lifestyle habits (including smoking and coffee drinking), and exposure to risk factors known as potentially affecting the susceptibility to spine disorders like family history of lumbar spine disorder (parents, brothers or sisters) [7, 14, 20], job physical demand for the majority of the working years (evaluated by score: 0 = sedentary; 1 = light; 2 = medium; 3 = heavy) [7, 14], exposure to vibrations as daily hours spent driving or as a passenger in motorized vehicles [7, 14], and weekly hours of physical exercise including athletic training and competitions.

### VDR-FokI polymorphism determination

Determination of *VDR*-FokI polymorphism was performed as described after extraction of genomic DNA from EDTA-venous blood samples [7, 33]. Genotypes were designated by a capital letter F allele (C nucleotide) for absence, and by a lowercase letter f allele (T nucleotide) for the presence of the FokI endonuclease restriction site, respectively [7]. Personnel performing genetic analysis were blinded to study subject data.

### Statistical analysis

Normally distributed continuous data were presented as mean ± standard deviation (SD). The Mann-Whitney *U*-test was used for comparison of continuous variables. The difference of proportions between specific genotypes and allele groups was assessed by Pearson’s χ^2^-test or Fisher’s exact test, as appropriate. Logistic regression was performed to evaluate the difference of genotypes and alleles between groups after adjustment for potential LBP confounders [4, 14, 15]: age, sex, BMI, ever smoking, exposure to vibrations, physical job demand, and weekly hours of physical exercise (including athletic training sessions and competitions). All tests were 2-sided. *P* values <0.05 were considered statistically significant, *p* < 0.10 values were considered a tendency. Statistical analysis was performed using the Statistical Package for Social Sciences (SPSS for Windows, SPSS Inc., Chicago, IL, USA).

## Results

### Sport activities

Table 1 shows sport disciplines practiced by the 60 study participants, of whom 16.7% were swimmers, followed by soccer players (11.7%), volleyball players (11.7%), rugby players (10.0%), and other disciplines.

**Table 1:** Sport activities of 60 Italian athletes

Sport discipline	*N* (%)
Swimming	10 (16.7)
Soccer	7 (11.7)
Volleyball	7 (11.7)
Rugby	6 (10.0)
Weight lifting	5 (8.3)
Track-and-field sports	5 (8.3)
Figure skating	4 (6.7)
Artistic gymnastics/competitive dancing	4 (6.7)
Basketball	3 (5.0)
Triathlon	3 (5.0)
Sailing	3 (5.0)
Discus throw	2 (3.3)
Martial arts	1 (1.7)

### Characteristics and habits of the study athletes and comparison between athletes with and without LBP

Out of 60 participants 24 (40%) had declared LBP episodes and 36 had no LBP episodes lifelong. Only 5 out of 24 LBP subjects reported having received medical assistance as outpatient for LBP. On average LBP positive subjects had 3.1 ± 3.0 LBP episodes lifelong. However, none of the subjects declaring the LBP symptom had a specific medical diagnosis [7], accordingly, they were non-specific LBP cases [9, 10].

Table 2 illustrates main characteristics and lifestyle habits of study athletes, and comparison between the 24 athletes with LBP and 36 athletes without LBP episodes. Thirty-eight percent (23/60) of participants were elite athletes with national and/or international competitive experience; the remaining athletes (61.7%, 37/144) were non-elite athletes competing at regional levels (mostly in Friuli-Venezia-Giulia Region, Northern Italy).

**Table 2:** Demographic and behavioral characteristics of 60 study athletes, comparison between the 24 athletes with LBP and 36 athletes without LBP episodes

Characteristic	All study athletes (*n* = 60)	LBP (*n* = 24)	No-LBP (*n* = 36)	*P* value
Sport activity, hours/week, mean ± SD	7.8 ± 3.3	7.4 ± 3.4	8.1 ± 3.2	0.16^b^
Elite (national/international experience), *n* (%)	23 (38.3)	7 (29.2)	16 (44.4)	0.23^c^
Non-elite, *n* (%)	37 (61.7)	17 (70.8)	20 (55.6)	0.23^c^
Female, *n* (%)	25 (41.7)	11 (45.8)	14 (38.9)	0.59^c^
Male, *n* (%)	35 (58.3)	13 (54.2)	22 (61.1)	0.59^c^
Age, years, mean ± SD	33.9 ± 13.3	31.6 ± 12.4	35.5 ± 13.8	0.26^b^
Age at first LBP episode, years, mean ± SD	23.1 ± 9.5	23.1 ± 9.5	–	–
Number of LBP episodes, mean ± SD	3.1 ± 3.0	3.1 ± 3.0	–	–
Weight, kg, mean ± SD	72.4 ± 14.7	74.1 ± 11.6	71.2 ± 16.5	0.33^b^
Height, m, mean ± SD	174.9 ± 8.3	176.2 ± 7.6	174.1 ± 8.8	0. 46^b^
BMI, kg/m^2^, mean ± SD	23.5 ± 3.5	23.8 ± 3.0	23.2 ± 3.9	0.29^b^
BMI ≥ 25, kg/m^2^, *n* (%)	15 (25.0)	6 (25.0)	9 (25.0)	1.00^c^
University/College, *n* (%)	40 (66.7)	19 (79.2)	21 (58.3)	0.094^c^
Married or separated/divorced, *n* (%)	25 (41.7)	8 (33.3)	17 (47.2)	0.28^c^
Current smoker, *n* (%)	7 (11.7)	3 (12.5)	4 (11.1)	1.00^d^
Ever (current or past) smoker, *n* (%)	17 (28.3)	7 (29.2)	10 (27.8)	0.91^c^
6 or more cigarettes/day ever smoker, *n* (%)	9 (15.0)	4 (16.7)	5 (13.9)	1.00^d^
11 or more cigarettes/day ever smoker, *n* (%)	6 (10.0%)	2 (8.3)	4 (11.1)	1.00^d^
Coffee drinkers, *n* (%)	50 (83.3)	22 (91.7)	28 (77.8)	0.29^d^
3 or more cups of coffee ^a^/day, *n* (%)	19 (31.7)	9 (37.5)	10 (27.8)	0.43^c^
Exposure to vibrations ≥2 h/day	22 (36.7)	13 (54.2)	9 (25.0)	0.022^c^
Physical job demand more than sedentary	27 (45.0)	11 (45.8)	16 (44.4)	0.92^c^
Physical job demand more than medium	12 (20.0)	6 (25.0)	6 (16.7)	0.52^d^
Family history of lumbar pathologies	6 (10.0)	5 (20.8)	1 (2.8)	0.033^d^

^a^Italian espresso cups of coffee

^b^P comparison of LBP and no-LBP by two-tailed Mann-Whitney *U*-test

^c^P comparison of LBP and no-LBP by two-tailed Pearson’s chi squared test

^d^P comparison of LBP and no-LBP by two-tailed Fisher’s exact test, as appropriate

Participants performed on average 7.8 ± 3.3 h (range 5–22 h) weekly of regular physical activity including training and competitions, they were 33.9 ± 13.3 years-old, most had university level education, the majority of subjects were not married, and all were of middle-class socioeconomic status. The average BMI was 23.5 ± 3.5 kg/m^2^; the majority of athletes (44/60, 73.3%) were in the normal weight range (BMI ≥18 and ≤25 kg/m^2^), 1 was an underweight female swimmer (BMI <18 kg/m^2^, 1.7%), and 15 were overweight athletes (BMI >25 kg/m^2^, 25.0%) of which 2 (both male rugby players) had BMI >30 kg/m^2^. Few athletes were current smokers (11.7%), had ever smoked (i. e. past or current smokers) were 28.3%, however, only 10.0% of study participants ever smoked 11 or more cigarettes per day in their lifetime.

As shown in Table 2, LBP athletes were not different from no-LBP athletes with regard to the majority of the studied parameters. In particular, athletes with LBP did no more hours of athletic training and competitions weekly, were not more frequently elite athletes, did not differ by sex, age, BMI, smoking status, and did not had a more physically intense job than athletes without LBP. However, LBP athletes were more frequently exposed to vibration for ≥2 h per day (OR = 3.54, 95% CI 1.18–10.7, *P* = 0.022), and more frequently had a family history of lumbar pathology (OR = 9.21, 95% CI 1.00–84.7, *P* = 0.033) than no-LBP athletes.

Table 3 shows frequencies of *VDR*-FokI genotypes and alleles in all 60 athletes and illustrates comparisons of values in 24 LBP cases versus 36 no-LBP controls. The homozygous FF genotype was present in 58.3% of LBP cases versus 27.8% of no-LBP controls with significant risky crude OR = 3.64 and adjusted OR = 5.78. The heterozygous Ff genotype was less frequent in LBP cases (41.7%) than in no-LBP controls (66.7%), with significant protective adjusted OR = 0.24. The homozygous genotype ff was found in none of LBP cases, and in 5.6% of no-LBP controls. The F allele had a frequency of 79.2% in LBP cases versus 61.1% in no-LBP controls, with significant risky crude OR = 2.42, and adjusted OR = 2.55, consequently the f allele was protective for LBP.

**Table 3:** VDR-FokI genotypes and alleles in 60 athletes, and comparison between 24 athletes with LBP episodes and 36 athletes without LBP episodes

VDR-FokI genotype or allele	All athletes, *N* = 60, *n* (%)	LBP, *N* = 24, *n* (%)	No-LBP, *N* = 36, *n* (%)	Crude OR (CI)	Crude *P* value	Adjusted^a^ OR (CI)	Adjusted^a^ *P* value
FF genotype	24 (40.0)	14 (58.3)	10 (27.8)	3.64 (1.22–10.8)	0.018^b^	5.78 (1.41–23.8)	0.015
Ff genotype	34 (56.7)	10 (41.7)	24 (66.7)	0.36 (0.12–1.04)	0.056^b^	0.24 (0.06–0.93)	0.039
ff genotype	2 (3.3)	0 (−)	2 (5.6)	–^c^	–^c^	–^c^	–^c^
F allele	82/120 (68.3)	38/48 (79.2)	44/72 (61.1)	2.42 (1.04–5.61)	0.037^b^	2.55 (1.02–6.43)	0.046
f allele	38/120 (31.7)	10/48 (20.8)	28/72 (38.9)	0.41 (0.18–0.96)	0.037^b^	0.39 (0.16–0.98)	0.046

^a^OR was adjusted by multivariate analysis for age, sex, BMI, ever smoking, exposure to vibrations, physical job demand, and weekly hours of physical exercise

^b^P comparison of LBP and no-LBP by two-tailed Pearson’s chi squared test

^c^OR not countable because one of the compared groups had zero subjects

## Discussion

At present, the scientific interest in the risk factors for LBP is increasing both for athletic performance and health implications [9, 10]. Further research is required in this field because discrepancies exist among studies especially regarding LBP prevalence, causes, and therapeutic strategies [9, 12, 35]. In addition, sex-specific studies are warranted to take into account sex differences in factors potentially modulating LBP [10, 14]. Some types of exercise seems to increase LBP prevalence rate [8–10], but studies comparing athletes and non-athletes do not always confirm this view [11, 36]. On the other hand, some evidence suggests that exercise is effective in preventing LBP [12, 35, 37].

The present observational study is the first to explore the relationship of non-specific LBP in athletes with *VDR*-FokI genotypes and alleles in a sample of 60 ethnically homogeneous white athletes practicing various sport disciplines. We found that the frequency of the homozygous FF genotype was higher in LBP athletes, with adjusted OR = 5.78. On the contrary, the Ff genotype was protective (adjusted OR = 0.24). Our findings highlighted that carriage of the F allele was a risk factor (adjusted OR = 2.55), whereas carriage of the f allele was protective for the development of LBP in athletes (adjusted OR = 0.39).

Genotype and allele frequencies in our LBP group (FF 58.3, Ff 41.7, ff 0, F allele 79.2%) were different to those reported in a study on 267 non-athletic patients with lumbar spine pathologies (FF 43.8, Ff 44.9, ff 11.2, F allele 66.3%) [7]. Our current findings in LBP athletes are similar to those found in a study of 64 Italian non-athletic patients who had discopathies (with or without disc herniation) (FF 57.8, Ff 34.4, ff 7.8, F allele 75.0, and f allele 25.0%). That study showed that the FF genotype and the F allele were risk factors (OR = 2.02, 95% CI 1.15–3.55, *P* = 0.015, and OR = 1.76, 95% CI 1.13–2.75, *P* = 0.012, respectively) by comparison to healthy non-athletic controls [7]. Moreover, a group of 87 Italian non-athletic patients with a medical diagnosis (by magnetic resonance imaging, MRI) of discopathies and/or osteochondrosis associated with disc herniation had FF rates of 56.3, Ff 36.8, ff 6.9, F allele 74.7, and f allele 25.3%. The odds ratio of FF was OR = 1.90, 95% CI 1.15–3.13, *P* = 0.012, and of F allele was OR = 1.74, 95% CI 1.17–2.57, *P* = 0.005 [7]. Unfortunately, the design of our present study was observational and, thus, we could not assess radiologically whether study athletes with the LBP symptom had a discopathy. Interestingly, Sward and colleagues [38] found that disc degeneration, defined as reduced disc signal intensity, was significantly more common in elite gymnasts athletes (75%) than in non-athletes (31%). There was also a significant correlation between back pain and reduced disc signal intensity. Moreover, a study by Ong and colleagues [39] examining 31 Olympic athletes of various disciplines with low back pain found disc degeneration (assessed as reduced disc signal intensity by MRI) in 62% of subjects and a prevalence of disc displacement of 58%. The authors suggested the opportunity for a change in the methods of training to minimize disc degeneration, particularly at the elite levels of sport [39].

Surprisingly, so far only 6 studies [40–45] have assessed *VDR*-FokI polymorphism in athletesprior to the current study. However, no previous studies have examined the association of *VDR*-FokI polymorphism with LBP in athletes. A study in 125 Italian soccer players determined frequencies of FF 51.2, Ff 34.4, ff 14.4, F allele 68.4, and f allele 31.6% [40]. In this study, the FF genotype was not related to different athletic performance, but it was associated with higher values of the ratio of body cell mass over fat-free mass (BCM/FFM) [40]. Similarly, a study in 80 Italian white male gymnasts participating at international competitions found no relationship between the *VDR*-FokI polymorphism and athletic performance [41]. In addition, 2 studies by Massidda and colleagues in male Italian soccer players found no correlation of *VDR*-FokI polymorphism with athletic performance [42], and no association with incidence or severity of musculoskeletal injury [43]. A study by Nakamura and colleagues [44] in 44 Japanese male competitive athletes of various sport disciplines found frequencies of FF of 50.0, and Ff 47.7%; FF carries when compared to Ff carriers had 7.7% greater lumbar spine bone mineral content (BMC) [44]. In contrast, a Brazilian study examining 46 adolescent soccer players (FF 41.3, Ff 47.8, ff 10.9, F allele 65.2, and f allele 34.8%) found higher total body mineral content and density in boys with Ff genotype compared to those with FF genotype [45].

By examining demographic and lifestyle characteristics, we observed that daily exposure for 2 or more hours to vehicle vibrations and family history of lumbar spine pathologies were risk factors for LBP in athletes. Similar results were previously observed for non-athletic patients with lumbar pathologies, especially those with discopathies and/or osteochondrosis associated with disc herniation [7, 14]. We have shown in this study that athletes with LBP were not older, were not more frequently males, had no higher BMI, were not more frequently smokers, and had no a more physically intense job than no-LBP controls. On the contrary of previous findings evidenced in non-athletic patients [7, 14]. Interestingly, hours of physical exercise did not differ between LBP and no-LBP athletes, conversely, non-athletic patients with lumbar pathologies did less leisure physical activity than healthy controls [7, 14].

In our study, BMI was not a risk factor for LBP. A study in elite junior divers [1] found that a higher BMI was a LBP risk factor for male but not female athletes. A recent comprehensive review on risk factors of non-specific LBP in athletes [9] found that there is moderate evidence for high body weight as a risk factor.

Interestingly, Moradi and colleagues [9] reported that there is insufficient evidence that amount of training per week, active years in sport, age, and sex are associated with LBP. We found that amount of weekly physical exercise, age and sex were not risk factors for LBP in athletes. Thus, our present findings seem to concur with most accumulated evidence of risk factors for LBP in athletes.

The effect of smoking on LBP in athletes was poorly investigated in previous studies [9], however, smoking in athletes is much less frequent and intense than in non-athletes [46].

Mechanisms leading to LBP in athletes still need to be clarified. Further studies with increased number of subjects, and also “ex vivo” research will be necessary to evaluate the biochemical pathways relating the vitamin D endocrine system to LBP.

Some evidence showed that FF genotype is associated with increased lumbar spine bone mineral density (BMD) [24, 44, 47]. Whether increased risk of LBP in athletes due to carriage of F allele is mediated by higher transcriptional activity of VDR and thus, higher biological efficacy of vitamin D in subjects with a specific genetic background needs further investigations. Future studies are warranted to assess if the observed increased frequency of FF genotype and F allele in LBP athletes is associated with up-regulation (or down-regulation) of specific genes responsive to VDR action and associated with lumbar spine pain. Interestingly recent studies showed that a wide variety of hundreds of genes can be affected by vitamin D [23, 26, 48]. Genome-wide studies indicated the existence of up to 2776 VDR binding sites in human genome [48]. Bioinformatic analysis has found 14,548 putative VDR binding sites; 16–21% of these sites are located at gene promoters [23]. VDR binding at VDREs may up- and down-regulate transcription of genes in a tissue specific manner, this is also modulated by epigenetic changes [23, 26].

Deeper understanding of biological pathways of vitamin D related to LBP may indicate whether vitamin D rich diet and/or supplementation should be recommended according to *VDR*-FokI polymorphism. Recently, dietary and vitamin supplementation were proposed to athletes having LBP [9]. To our knowledge none so far suggested specifically vitamin D supplementation to athletes with LBP. Future studies will be necessary to assess this issue of personalized medicine [28, 49, 50].

It is generally recognized that LBP can negatively affect athletic performance and recovery. Assessing the predisposing and protective factors for LBP in athletes may be complicated by several confounding factors, among these *VDR*-FokI polymorphism may constitute a major confounder as shown by our present findings.

There are limitations in our study. We studied adult athletes, and thus, we cannot generalize these results to younger or older athletes or athletes with different ethnic backgrounds. Sport activities of athletes were heterogeneous ranging from aerobic to mixed aerobic-anaerobic and to anaerobic activity, and from elite to non-elite competing level. We included several types of sport disciplines but some other disciplines like tennis, skiing, golf etc. were not assessed. However, we did not prove a LBP association with elite status and hours of physical exercise per week. In our study we examined non-specific LBP thus our results cannot address other kinds of LBP in athletes. In our study the confidence intervals were somewhat large; however, significant results were obtained after multivariate adjustments including several confounders. Finally, we evaluated LBP by a questionnaire and we did performed radiological investigations.

Strengths of the present study include assessment of LBP and *VDR*-FokI polymorphism in a homogeneous ethnic group of competitive athletes, evaluation of demographic, social, lifestyle characteristics and family history, and stringent inclusion and exclusion criteria.

Further studies have to be carried out to expand our observations including larger numbers of athlete practising different sport disciplines and to better assess biochemical pathways related to vitamin D’s effects on LBP in athletes.

## Conclusion

LBP in competitive athletes is under study, but risk factors are still to be better investigated. This is the first study (to our knowledge) demonstrating that a genetic trait of the vitamin D system is associated with LBP in athletes. We also investigated lifestyle habits of study athletes. We have highlighted for the first time that some (although not all) risk factors for LBP in the general non-athletic population are also risk factors in athletes. We suggest that among LBP risk factors examined in athletes, daily exposure to vehicle vibrations and family history of lumbar pathologies should be investigated. Identification of genetic and non-genetic risk factors for LBP could be used by each athlete to personalize training and lifestyle habits [51]. Further studies with more defined LBP diagnosis by use of instrumental imaging techniques are needed.

## Additional file

Additional file 1: Subject N.; LBP_bin, Fok genotype, F1_1_FF, F1_2_Ff, F2_2_ff . Description of data: list of subjects with indication of LBP yes or no, and FokI genotype for each subject. (PDF 16 kb)

## Abbreviations

BCM: Body cell mass
BMC: Bone mineral content
BMI: Body mass index
FFM: Fat free mass
LBP: Low back pain
SNP: Single nucleotide polymorphism
SPSS: Statistical package for social sciences
TFIIB: Transcription factor IIB
VDR: Vitamin D receptor
VDRE: Vitamin D response element
