# Supplementary material for: Low back pain and FokI (rs2228570) polymorphism of vitamin D receptor in athletes
Source: BMC Sports Sci Med Rehabil. 2017 Feb 7;9:4. doi: 10.1186/s13102-017-0069-x (PMC5294822; doi:10.1186/s13102-017-0069-x)
Supplement: Additional file 1: — Subject N.; LBP_bin, Fok genotype, F1_1_FF, F1_2_Ff, F2_2_ff . Description of data: list of subjects with indication of LBP yes or no, and FokI genotype for each subject. (PDF 16 kb) [file 13102_2017_69_MOESM1_ESM.pdf]

Subject.N.	LBP_bin	Fok genotype	F1_1_FF	F1_2_Ff	F2_2_ff
1	0	1.2	0	1	0
2	0	1.2	0	1	0
3	1	1.2	0	1	0
4	1	1.2	0	1	0
5	0	1.1	1	0	0
6	0	1.2	0	1	0
7	1	1.1	1	0	0
8	0	1.2	0	1	0
9	0	1.2	0	1	0
10	0	1.1	1	0	0
11	1	1.1	1	0	0
12	1	1.1	1	0	0
13	1	1.1	1	0	0
14	1	1.2	0	1	0
15	1	1.1	1	0	0
16	1	1.1	1	0	0
17	1	1.1	1	0	0
18	1	1.2	0	1	0
19	0	1.2	0	1	0
20	0	1.2	0	1	0
21	1	1.1	1	0	0
22	1	1.2	0	1	0
23	1	1.2	0	1	0
24	0	1.2	0	1	0
25	0	1.2	0	1	0
26	0	1.2	0	1	0
27	1	1.1	1	0	0
28	0	1.1	1	0	0
29	0	1.1	1	0	0
30	0	1.2	0	1	0
31	0	1.2	0	1	0
32	0	1.1	1	0	0
33	0	1.2	0	1	0
34	0	2.2	0	0	1
35	0	1.2	0	1	0
36	0	1.2	0	1	0
37	0	1.1	1	0	0
38	0	1.2	0	1	0
39	0	1.2	0	1	0
40	0	1.2	0	1	0
41	0	1.2	0	1	0
42	1	1.2	0	1	0
43	1	1.2	0	1	0
44	0	1.2	0	1	0
45	1	1.2	0	1	0
46	1	1.1	1	0	0
47	0	1.1	1	0	0
48	0	1.2	0	1	0
49	0	1.1	1	0	0
50	0	1.1	1	0	0
51	0	2.2	0	0	1

52	1	1.1	1	0	0
53	1	1.1	1	0	0
54	1	1.2	0	1	0
55	0	1.1	1	0	0
56	0	1.2	0	1	0
57	0	1.2	0	1	0
58	1	1.1	1	0	0
59	1	1.1	1	0	0
60	0	1.2	0	1	0
